# The toll of 24/7 societal demands: the brain health risks of shift work

**DOI:** 10.1093/sleep/zsae134

**Published:** 2024-06-15

**Authors:** Masoud Tahmasian, Vincent Küppers

**Affiliations:** Institute of Neuroscience and Medicine, Brain and Behaviour (INM-7), Research Center Jülich, Jülich, Germany; Department of Nuclear Medicine, University Hospital and Medical Faculty, University of Cologne, Cologne, Germany; Institute of Systems Neuroscience, Medical Faculty and University Hospital Düsseldorf, Heinrich Heine University, Düsseldorf, Germany; Institute of Neuroscience and Medicine, Brain and Behaviour (INM-7), Research Center Jülich, Jülich, Germany; Department of Nuclear Medicine, University Hospital and Medical Faculty, University of Cologne, Cologne, Germany; Institute of Systems Neuroscience, Medical Faculty and University Hospital Düsseldorf, Heinrich Heine University, Düsseldorf, Germany

In our modern societies, 24 hours a day, 7 days a week (24/7) public services are needed. The International Labor Organization and Eurofound study covering approximately 1.2 billion workers in Europe, Asia, and the Americas demonstrated that half of the shift workers engage in rotating shifts, and between 10% and 20% of them have night shifts at least once a month [1]. Circadian desynchrony, abnormal sleep–wake patterns, and daily dysfunction due to sleep disturbance are risk factors for chronic diseases such as cardiometabolic conditions (e.g. sudden cardiac death, myocardial infarction, obesity, and type 2 diabetes), breast, prostate, and colorectal cancers, as well as sleepiness-related vehicle accidents in shift workers [1–4]. Thus, shift work paints a worrisome health picture of medical staff, factory and transport workers, police officers, firefighters, and other first responders. Meta-analyses demonstrated that shift workers are at higher risk for dementia [5] and poor mental health, mainly depressive symptoms, which are more prevalent in female shift workers [6]. Despite its high prevalence [7, 8], noticeable cost [9], and significant impact on daytime productivity [10, 11] and brain and body health [2–6], our understanding of the neurobiological mechanisms by which such sleep disturbance and circadian desynchrony affect the brain and behavior is limited.

Recently, Song et al. assessed the link between sleep quality, hippocampal volume, and psychomotor speed in a sample of firefighter shift workers with good (*n* = 59) or poor (*n* = 41) sleep quality and 106 matched controls [12]. The authors found that the shift workers experiencing poor sleep quality had reduced hippocampal volumes compared to those with good sleep quality and non-shift workers. More reduction in hippocampal volume was linked with an extended shift work history. Interestingly, psychomotor speed was impaired in shift workers with poor sleep compared to non-shift workers, and only in this group was psychomotor speed linked with more hippocampal atrophy (particularly in the cornu ammonis). Their results highlighted the hippocampus as a vulnerable brain area for sleep disturbance in chronic shift workers, as other brain areas involved in psychomotor speed processing (e.g. the motor-premotor and parietal cortex) were less affected by shift work-related sleep disturbance.

The neuroimaging studies that have examined the neurobiological underpinnings of shift work have largely found divergent results. Typically, those were based on relatively small samples and mostly had cross-sectional designs. Brain atrophy in the postcentral gyrus, paracentral lobule, and superior temporal gyrus have been reported in night-shift nurses compared to day-working nurses [13], as well as in the pontomesencephalic tegmentum volume that was related to poor sleep quality [14]. However, another study did not observe abnormality in cortical thickness, surface area, or subcortical volumes between shift workers and non-shift workers [15]. The white matter integrity of the anterior cingulate cortex (ACC) was altered in shift workers and was linked with poor sleep quality [16]. Rotating shift workers represent brain functional alterations as well e.g. lower resting-state functional connectivity between the ACC and right insula, and between the right anterior insula and right superior parietal lobule, as well as higher functional connectivity between the ACC with the left occipital lobe, right superior frontal gyrus, and the supplementary motor area, highlighting the role of the salience network dysfunction in shift workers [17]. A multimodal study, however, could not identify graph-based functional connectivity, cortical thickness, and gray matter volume alterations in night-shift workers and also did not observe any association between night-shift work and cognition [18]. These divergent neuroimaging findings and general concerns about sample size to identify robust associations between brain and cognitive/phenotypes highlight the need for further replications [19]. Neuroimaging meta-analyses, multi-center data-sharing, and large-scale data analyses of deep phenotypes can help to overcome shortcomings of single, medium-powered studies, and to identify robust findings [19, 20].

Psychomotor performance is a crucial component of daily functioning and work-related tasks, and shift work has an adverse impact on psychomotor and cognitive performance [12]. A reduction in performance has been observed in numerous occupational groups during and immediately following shift work. However, the evidence regarding the long-term effects of cognitive deficits is heterogeneous [21] Differences were observed between non-shift workers and current and recent shift workers, but not between past shift workers (not working shifts for more than 5 years) and non-shift workers [22]. These findings indicate that cognitive differences may be transient and potentially reversible. Slower psychomotor performance has been associated with both shorter and longer sleep duration [23]. One possible explanation for differences in psychomotor performance could be sleep disturbances related to shift work, as suggested by Song et al. [12]. Therefore, it remains to be explored whether potential psychomotor and cognitive performance deficits, as well as mental health, may be causally linked to structural and/or functional brain changes due to shift work. Another question to be addressed is why some shift workers develop sleep disturbance, leading to brain, and behavioral maladaptation, as brain and psychomotor findings were primarily observed in shift workers with poor sleep, reflecting the mediatory role of sleep disturbance [12].

The study by Song et al. found interesting hippocampal alterations in relation to sleep quality in shift workers; however, the generalizability of their findings is limited by its small sample size in one occupation, focusing on structural brain data only, and the simple correlational approach using a cross-sectional design. Thus, future studies should use (1) a more extensive data, which is essential to identify a reproducible brain-behavior association [20]; (2) longitudinal design to assess the long-term impact of shift work on brain health; (3) deeply phenotyped individuals to find the interplay between sleep disturbance and various cognitive, psychomotor, and mental health factors[24]; (4) cross-country and multi-center data as sleeping patterns are divergent in different countries [25, 26]; (5) multimodal neuroimaging data to capture brain abnormalities at both structural and functional levels; (6) diverse samples across different occupations, e.g. highly demanding jobs, including frontline doctors, and nurses compared to other jobs that permit adequate sleep during shift [10, 27]; (7) further studies should test the roles of “biopsychosocial” factors, including genetic, circadian dysrhythmia (e.g. due to suprachiasmatic nucleus dysfunction), lifestyle, environmental, and psychological factors such as emotion regulation on brain health to identify why only a group of shift workers develop sleep disturbance. Finally, despite the uncertainty surrounding the cognitive and neurobiological changes associated with shift work, it is evident that we need novel and feasible strategies, such as more physical activity [28] and better working hour policies, in order to minimize the [12] consequences of shift work for brain and body health and reduce this impact in our societies.
